# Associations between a health-promoting lifestyle and quality of life among adults with beta-thalassemia major

**DOI:** 10.4178/epih.e2016050

**Published:** 2016-11-15

**Authors:** Aghbabak Maheri, Roya Sadeghi, Davoud Shojaeizadeh, Azar Tol, Mehdi Yaseri, Mojtaba Ebrahimi

**Affiliations:** 1 Department of Health Education and Promotion, School of Public Health, Tehran University of Medical Sciences, Tehran, Iran; 2 Department of Epidemiology and Biostatistics, School of Public Health, Tehran University of Medical Sciences, Tehran, Iran; 3 Zafar Adult Thalassemia Clinic, Iranian Blood Transfusion Organization, Tehran, Iran

**Keywords:** Quality of life, Life style, Adult, Beta-thalassemia

## Abstract

**OBJECTIVES:**

A health-promoting lifestyle (HPL) is a factor that affects the quality of life (QoL) in patients with beta-thalassemia (β-thalassemia). Due to the lack of studies of this issue, this study aimed to determine the association between HPL and QoL among adults with β-thalassemia.

**METHODS:**

This cross-sectional (descriptive-analytic) study was conducted among 389 adult patients with β-thalassemia in Tehran, Iran. The research instrument included a questionnaire consisting of three parts: demographic items, the Short-Form Health Survey and the Health-Promoting Lifestyle Profile. The data were analyzed using SPSS version 23.0. The results were considered significant at the conventional p<0.05 level.

**RESULTS:**

The mean age of the participants was 30.2±8.3 years. The mean score of the HPL dimensions was 127.28±21.53, and the mean score of the QoL domains was 61.44±23.38. The highest and the lowest mean scores of the HPL dimensions were found for spiritual growth (23.96±5.74) and physical activity (11.32±3.95), respectively. The QoL scores in all three domains (total, physical component summary score, and mental component summary score) were moderate. Health responsibility, physical activity, spiritual growth, and interpersonal relations were significant predictive factors of QoL in adults with β-thalassemia; these four dimensions explained 37.9% of the variance in QoL.

**CONCLUSIONS:**

QoL and HPL were not at acceptable levels among patients with thalassemia. Therefore, educational interventions emphasizing spiritual growth, physical activity, and interpersonal relations are necessary for patients with thalassemia.

## INTRODUCTION

The World Health Organization (WHO) has recognized thalassemia, which is found in more than 60 countries, as the most prevalent genetic blood disorder globally [1]. Studies have reported that 3% to 10% of the population of the world carries a thalassemia gene [1]. Approximately 200,000 patients with thalassemia have been documented in the world, and each year approximately 60,000 people are added to this number [2,3].Thalassemia can occur in almost all races, but areas with high incidence (known as the thalassemia belt) are found in the Mediterranean region (Italy, Spain, Portugal, Greece, parts of Russia, and Cyprus), north and west Africa, the Middle East (Saudi Arabia, Iran, Turkey, and Syria), the Indian subcontinent (India and Pakistan), southeast Asia (Bangladesh, Indonesia, Thailand, Malaysia, and southern China), and South America [4]. Approximately 240 million people worldwide carry beta-thalassemia (β-thalassemia) [2]. β-thalassemia is a global health problem in the Mediterranean region, Middle East, Indian subcontinent, and southeast Asia [3,5]. Iran is a country located in the thalassemia belt, with an average thalassemia gene prevalence rate of 4% (approximately 3 million people) [2,6]. In Iran, approximately 26,000 people have thalassemia and approximately 800 people are added to this number annually [2].

Achievements in biomedical sciences and technology have led to significant improvements in health-related quality of life (QoL) for thalassemia patients. Enhanced survival in these patients poses issues related to their QoL [3]. Some of the major clinical and psychological aspects of thalassemia are expected to have an effect on the QoL of patients with thalassemia, including (1) having a chronic condition and the subjective feeling of being different; (2) changes in appearance, such as bone deformities and short stature, which lead to a poor self-image; (3) treatment, which involves frequent hospital visits for transfusions and iron chelation therapy; (4) delayed or absent sexual development and problems related to fertility; (5) complications such as heart disease, bone disease, diabetes, and infections; (6) uncertainties about the future and problems related to long-term planning [7,8].

Studies have emphasized that, in addition to controlling the symptoms of the disease, it is very important to improve QoL as part of the treatment of a chronic disease such as thalassemia [3,9]. Additionally, studies have shown that patients with thalassemia reported lower mean scores of QoL than healthy subjects [8,9]. With access to better methods of blood transfusions and iron chelation therapy, better management of complications and supportive care are available for patients with thalassemia, and they now have a near-normal lifespan with a good QoL [10]. Some studies have evaluated the QoL of patients with thalassemia, but the factors affecting QoL in these patients are largely unknown [3,11,12]. Understanding the factors associated with QoL in patients with thalassemia has direct implications for the design and implementation of clinical, counseling, and supportive interventions to enhance treatment outcomes and QoL among these patients [12].

One of the primary objectives of the Healthy People 2010 initiative (US Department of Health and Human Services, 2006), was to improve the QoL of adults with chronic diseases. Preserving a satisfactory QoL is considered to be a major objective of nursing care. Health-promoting behavior, particularly when integrated into a healthy lifestyle that pervades all aspects of living, should result in improved health, enhanced functional ability, and better QoL at all stages of development [13]. Studies show that a health-promoting lifestyle (HPL) is a factor affecting QoL [13-15]. A healthy lifestyle consists of six domains, including physical activity, nutrition, health responsibility, spiritual growth, interpersonal relations, and stress management [16]. Since QoL in patients with thalassemia is affected by lifestyle [17], and given the lack of studies of this issue, this study aimed to determine the association between HPL and QoL in adults with β-thalassemia.

## MATERIALS AND METHODS

This cross-sectional (descriptive-analytic) study was conducted between March 2015 and July 2016. The participants were adult patients with thalassemia major who were referred to thalassemia centers at hospitals affiliated with the Tehran University of Medical Sciences (TUMS), Iran University of Medical Sciences (IUMS), or the Iranian Blood Transfusion Organization (IBTO) in Tehran, the capital of Iran. The inclusion criteria were being more than 18 years old, having a definitive diagnosis of β-thalassemia major, providing informed consent to participate in the study, and having no other serious physical or mental health problems. A total of 389 patients were selected using stratified random sampling. Among nine thalassemia centers at hospitals affiliated with TUMS, IUMS, and IBTO, the centers that had more than 50 adult patients with thalassemia (Zafar Adult Thalassemia Clinic, Baharloo Hospital, Ali Asghar Children Hospital, and Children’s Medical Center Hospital) were identified as strata. Proportional to the number of patients in each stratum, the required samples were then selected randomly within in each center. We obtained the sampling frame of each center from the list of patients of each center. Simple random sampling was then used to select the required sample within each center. Random selection was performed using the SPSS select case menu. Ethical approval was obtained from TUMS (IR.TUMS.REC.1394. 1153).

The research instrument included a questionnaire consisting of three parts. The first component addressed demographic characteristics (11 items). The second component was the Persian version of the Short-Form Health Survey (SF-12) [18], including two main domains (the physical component summary [PCS] and the mental component summary [MCS]), as well as eight scales for assessing the eight dimensions of physical functioning (2 items), physical role (2 items), social role (1 item), emotional role (2 items), bodily pain (1 item), general health (1 item), vitality (1 item), and mental health (2 items). Scores on this instrument range from 0 to 100, where 0 indicates the worst condition and 100 indicates the best possible condition. The SF-12, as a shorter alternative to the SF-36, is widely used in health outcome surveys [18].Third, the Health-Promoting Lifestyle Profile (HPLP-II) was designed by Walker et al. [19] in 1978. In the current study, the validated and reliable Persian version of the HPLP-II [20] was used. The original version of this questionnaire consists of 52 items that measure HPL in six dimensions: nutrition (9 items), physical activity (8 items), health responsibility (9 items), stress management (8 items), interpersonal relations (9 items), and spiritual growth (9 items). These questions are given responses on a 4-point Likert scale (never, sometimes, often, and routinely). Overall, the score for HPL and the behavioral aspects was calculated using the mean of the responses for all 52 items and for each dimension (8 or 9 items). The scores ranged from 52 to 208 [20].

In this study, the dimensions of nutrition and physical activity were changed in accordance with the specific circumstances of thalassemia patients. For this reason, the validity and reliability of the final version of the data collection instruments were assessed again. Content validity was checked by five academic members of TUMS who were experts in the fields of health education and promotion, and five physicians who had worked in thalassemia clinics. The Cronbach alpha was calculated and the test-retest method was used to determine the reliability (with a 2-week interval for 20 patients). The intraclass correlation coefficient (ICC) for the SF-12 scale was 0.74 (95% confidence interval [CI], 0.45 to 0.89) and for the HPLP-II scale it was 0.92 (95% CI, 0.82 to 0.97). The alpha coefficient for the SF-12 was 0.88, and for the HPLP-II it was 0.89. These 20 patients were excluded from the main study.

Data were analyzed using SPSS version 23.0 (IBM Corp., Armonk, NY, USA) through descriptive methods (frequencies, percentage, mean, and standard deviation [SD]) and Pearson correlation coefficient multilevel linear regression. Parametric tests were used according to the kurtosis, skewness, and normalization of the data. When the study participants are clustered within a team or center, they are expected to have a within-center (or within-team) correlation in their response, which means that the results within a center are generally more homogenous than results obtained from multiple centers. This should be accounted for in the calculation of the standard error of estimations, so we used a multilevel model. These models can take the hierarchical structure of data into account by considering a random effect in addition to the fixed effect in their formulas. Centers in this model introduce a random effect, and in this way difference between centers were considered. Samples within each center shared a single or multiple random effect(s) that was/were different from center to center. The distribution of this random effect was univariate or multivariate normal with mean 0 and a variance (or covariance matrix) that could change from a random effect to another one [21]. Results were considered significant at the conventional level of p<0.05.

## RESULTS

### Participant characteristics

The demographic characteristics of the participants are summarized in Table 1. The mean age of the 389 adults with β-thalassemia was 30.2±8.3 years. Most of the participants (56.0%) were drawn from the Zafar Adult Thalassemia Clinic. Almost 46.0% were male, 67.1% were single, and 44.0% had a high-school diploma.

### Health-promoting lifestyle and quality of life

The mean and SD of the HPL and QoL scores are presented in Table 2. The mean total HPL score in participants was 127.28±21.53, while the mean scores in the dimensions of health responsibility, physical activity, nutrition, spiritual growth, interpersonal relations, and stress management were 23.63±5.06, 11.32±3.95, 27.21±4.96, 23.96±5.74, 23.95±5.20, and 17.18±3.77, respectively. The mean total QoL score, physical component summary score, and mental component summary score of the participants were 61.44±23.38, 60.14±26.26, and 62.73±24.63, respectively. Spiritual growth (66.5%) and interpersonal relations (66.5%) were the highest scores, and physical activity (47.2%) had the lowest score in the HPL dimensions of the adults with thalassemia. Participants achieved 61.2% of the total possible HPL score. The scores of QoL in all three domains (total, physical component summary score, and mental component summary) were moderate.

### Association between health-promoting lifestyle and quality of life

The Pearson correlation coefficient was used to describe the magnitude and direction of the associations of QoL with HPL and its dimensions. The results of this study showed a positive and statistically significant correlation between QoL and total HPL and its dimensions among adults with thalassemia (p< 0.001) (Table 3).

### Regression analyses of health-promoting lifestyle and its dimensions on quality of life

The regression coefficients for the final model are shown in Tables 4 and 5. Multilevel linear regression showed that four dimensions of HPL (health responsibility, physical activity, spiritual growth, and interpersonal relations) were significant predictive factors of QoL in adults with thalassemia. These four dimensions explained 37.9% of the variance of QoL, while 62.1% remained unexplained. Spiritual growth (standardized β=0.374; p<0.001), physical activity (standardized β=0.242; p<0.001), and interpersonal relations (standardized β=0.185; p<0.001) were independently and positively associated with QoL. However, health responsibility was independently and negatively associated with QoL (standardized β=-0.113; p<0.05). The strongest predictor of the QoL score in the adults with thalassemia was the spiritual growth score (standardized β=0.374). After adjustment for demographic characteristics, including age, sex, marital status, educational level, and employment status, the main results of the regression model did not change significantly.

## DISCUSSION

Based on results of this study, the QoL among the participants was moderate in the three domains under study (total QoL score, physical component summary score, and mental component summary score). However, this result is not very good, and the participants in this study had lower mean QoL scores than have been reported in surveys of healthy Iranian individuals. For example, in a study conducted by Montazeri et al. [22] on healthy Iranian individuals, the mean scores for QoL dimensions ranged from 65.6±41.4 to 85.3±20.8 (the lowest score was for emotional role dimension [65.6±41.4] and the highest score was for physical functioning dimension [85.3±20.8]). Additionally, in a study conducted by Fallahzadeh et al. [23] among Iranian university students, the mean QoL score was 64.2±10.2. The results of these studies indicate that the patients with thalassemia in our study had a lower QoL than healthy individuals. Such a large difference in the QoL of healthy individuals and patients with thalassemia is not surprising, because many factors have a negative impact on QoL in patients with thalassemia, such as having a chronic condition and the subjective sensation of being different; frequent hospital visits for transfusions and iron chelation therapy, delayed or absent sexual development and problems related to fertility, and complications such as heart disease, bone disease, diabetes, and infections [7,8].

Similar to the findings of this study, Haghpanah et al. [24], and Safizadeh et al. [25] reported lower QoL scores in patients with thalassemia in Iran. Compared with the results of a study that was carried out by Safizadeh et al. [25] of the QoL of thalassemia patients aged 16 years and older, higher mean scores of QoL were found in this study, whereas our findings were lower than the results of Haghpanah et al. [24] and Amani et al. [26]. The results of this study were primarily compared with the results of other studies conducted in Iran, because culture is an independent factor that influences the QoL of thalassemia patients [24,27]. However, the results of this study were also compared with those of foreign studies, and our findings are in agreement with the results of some of them [27]. Since the QoL of patients with thalassemia was not at an acceptable level, educational interventions are necessary to enhance the QoL of patients with thalassemia. Health education and promotion can be powerful tools for improving the QoL for all members of the population, including patients with thalassemia; therefore, applying these techniques seems reasonable due to their influence on health-promoting behaviors and improvements in QoL [28, 29].

The HPL scores obtained in this study were slightly lower than moderate [30-32]. Many studies have been performed to assess the HPL of healthy individuals, and have found moderate mean HPL scores [32,33]. The results of our study indicated that the extent of HPL among patients with thalassemia was not at an acceptable level. Therefore, conducting health education programs to improve HPL and establishing counseling centers for education about HPL for patients with thalassemia is necessary. For this reason, educational and health promotion interventions based on predictors of QoL are recommended to improve HPL in thalassemia patients.

The participants in this study showed relatively high scores for spiritual growth, interpersonal relations, and health responsibility, and lower scores for nutrition and stress management. The lowest score corresponded to physical activity. In a study of couples referred to an infertility clinic, the highest scores were obtained on the nutrition, spiritual growth, and interpersonal relations dimensions, while the lowest scores were for the health responsibility, physical activity, and stress management dimensions [34]. Similar to findings of this study, a study conducted among academic staff showed that participants obtained relatively high scores for spiritual growth, interpersonal relations, and nutrition, but relatively low scores for health responsibility and stress management, and the lowest score corresponded to physical activity [32]. Since this study found that the mean score for physical activity was lower than the scores of other dimensions, attention should be directed towards addressing both the challenges of individuals and providing supportive relationships for behavioral changes (physical activity) when designing interventions.

In this study, we found that spiritual growth, physical activity, interpersonal relations, and health responsibility were significant predictive factors of QoL in adults with thalassemia. In a study conducted by Şenol et al. [14], spiritual growth was a significant predictive factor of QoL. Likewise, Rakhshani et al. [31] concluded in their study that three dimensions of HPL (physical activity, spiritual growth, and stress management) were significant predictive factors of QoL. In this study, spiritual growth, physical activity, and interpersonal relations were independently and positively associated with QoL, while health responsibility was independently and negatively associated with QoL. Therefore, aspects of spiritual growth, physical activity, and interpersonal relations must be included in health interventions in order to improve QoL in patients with thalassemia. To achieve these goals, it is recommended to make use of the capabilities of health education and promotion experts and psychologists in thalassemia treatment centers. One of the strengths of this study was that it assessed the associations between the dimensions of HPL and QoL in patients with thalassemia. Due to the lack of similar studies in this field, this study can be considered as a source of guidance and a basis for future research, especially for interventional studies. However, this study has some limitations. First, in cross-sectional studies, the existence of alternative explanations for the results due to confounding must be carefully considered. Second, the results of this study were compared with the results of studies conducted among other groups, such as infertile couples, academic staff, the elderly, and nursing home residents. Third, self-reported data were collected, and it is possible that the results may not reflect an exact estimation. Fourth, due to the nature of the study sample, it is possible that thalassemia patients in cities other than Tehran show different trends than those sampled in this study. Fifth, the results of this study were compared with the results of other studies that were conducted in Iran, because thalassemia is endemic in Iran and culture is an independent factor that influences the QoL of thalassemia patients.

In general, the results of our study indicate that the QoL and HPL of patients with thalassemia were not at acceptable levels. Spiritual growth, physical activity, and interpersonal relations were found to be relatively strong predictors of QoL in this study. Therefore, it is necessary to implement health education programs with an emphasis on spiritual growth, physical activity, and interpersonal relations, as well as to establish counseling centers for the education of patients with thalassemia regarding HPL.

## Figures and Tables

**Table 1.** Demographic characteristics of the study participants (n=389)

Variable	Frequency	%
Thalassemia center		
Children's Medical Center Hospital	78	20.1
Ali Asghar Children Hospital	53	13.6
Zafar Adult Thalassemia Clinic	218	56.0
Baharloo Hospital	40	10.3
Age (mean±SD, yr)	30.2±8.3	
Sex		
Male	179	46.0
Female	210	54.0
Marital status		
Single	261	67.1
Married	128	32.9
Educational level		
Illiterate	5	1.3
No high-school diploma	51	13.1
High-school diploma	171	44.0
University	162	41.6
Employment status		
Household	60	15.4
School student	11	2.8
University student	44	11.3
Government employee	33	8.5
Self-employed	167	42.9
Unemployed	74	19.0
Total	389	100.0

SD, standard deviation.

**Table 2.** Mean scores of the HPL and quality of life scales

Variable	Mean±SD	Scale range^1^	Min-Max^2^	Mean score (out of 100%)
Health responsibility	23.63±5.06	9, 36	10-36	65.6
Physical activity	11.32±3.95	6, 24	6-24	47.2
Nutrition	27.21±4.96	11, 44	16-44	61.8
Spiritual growth	23.96±5.74	9, 36	9-36	66.5
Interpersonal relations	23.95±5.20	9, 36	11-36	66.5
Stress management	17.18±3.77	8, 32	8-32	53.7
Total HPL score	127.28±21.53	52, 208	70-205	61.2
Physical component summary	60.14±26.26	0, 100	0-100	60.1
Mental component summary	62.73±24.63	0, 100	0-100	62.7
Total SF-12 score	61.44±23.38	0, 100	2.08-100	61.4

HPL, health-promoting lifestyle; SD, standard deviation; SF-12, Short-Form Health Survey; Min, minimum; Max, maximum.

1 The lowest and highest values that can be obtained in the original scale.

2 The lowest and highest values that were obtained in this study.

**Table 3.** Pearson correlations between health-promoting lifestyle (HPL) dimensions and quality of life

HPL dimensions	r	p-value
Health responsibility	0.212	<0.001
Physical activity	0.455	<0.001
Nutrition	0.237	<0.001
Spiritual growth	0.553	<0.001
Interpersonal relations	0.461	<0.001
Stress management	0.420	<0.001
Total HPL score	0.521	<0.001

**Table 4.** Predictive factors of quality of life among the participants as determined by multilevel linear regression analysis

HPL dimensions	R^2^	Unstandardized coefficients		β	95% CI		p-value
		B	SE		Lower	Upper	
Health responsibility	0.045	-4.701	2.18	-0.113	-8.98	-0.42	0.03
Physical activity	0.211	8.601	1.83	0.242	5.01	12.19	<0.001
Nutrition	0.211	-0.736	2.72	-0.014	-6.08	4.61	0.79
Spiritual growth	0.359	13.709	2.07	0.374	9.64	17.78	<0.001
Interpersonal relations	0.379	7.474	2.21	0.185	3.12	11.83	<0.001
Stress management	0.379	0.426	2.95	0.009	-5.38	6.23	0.88

HPL, health-promoting lifestyle; SE, standard error; CI, confidence interval.

**Table 5.** Predictive factors of quality of life adjusted for demographic characteristics^1^ among the participants according to a multilevel linear regression analysis

HPL dimensions	Unstandardized coefficients		β	95% CI		p-value
	B	SE		Lower	Upper	
Health responsibility	-5.614	2.20	-0.135	-9.94	-1.29	0.01
Physical activity	7.172	1.87	0.202	3.50	10.85	<0.001
Nutrition	2.420	2.79	0.047	-3.07	7.91	0.39
Spiritual growth	12.341	2.07	0.337	8.28	16.40	<0.001
Interpersonal relations	6.267	2.21	0.155	1.93	10.61	0.005
Stress management	0.547	2.90	0.011	-5.16	6.25	0.85

PL, health-promoting lifestyle; SE, standard error; CI, confidence interval.

1 Adjustment was performed for age, sex, marital status, educational level, and employment status.
